# Temperatures Lower than Preferred Ones Maintain DNA Integrity and Sperm Quality of *Lepidophyma gaigeae* (SQUAMATA: XANTUSIIDAE)

**DOI:** 10.3390/ani15121784

**Published:** 2025-06-17

**Authors:** Daniel Uriostegui Escoto, Fausto Roberto Méndez de la Cruz, Mario García Lorenzana, Yolanda López Ramírez, Edith Arenas Ríos

**Affiliations:** 1 Doctorado en Ciencias Biológicas y de la Salud, Universidad Autónoma Metropolitana, Iztapalapa, Ciudad de México C.P. 09340, Mexico; yols_yj89@hotmail.com; 2 Departamento de Zoología, Instituto de Biología, Universidad Nacional Autónoma de México, A.P. 70515, Coyoacán C.P. 04510, Mexico; faustomendez6@gmail.com; 3 Departamento de Biología de la Reproducción, Universidad Autónoma Metropolitana, Unidad Iztapalapa, Ciudad de México C.P. 09340, Mexico; lorenzanagm@gmail.com

**Keywords:** sperm, lizards, behavior, thermal effect, reproduction, DNA fragmentation

## Abstract

The global increase in temperature has directly impacted the environment and reproduction of ectothermic organisms, leading to defects in processes that intervene in sperm quality and maturation. The present study evaluates the effect of temperature on sperm quality of the lizard *Lepidophyma gaigeae* in the surroundings of Landa de Matamoros, Querétaro, México. As a species under special protection, only 12 male individuals were captured in December 2020 with 4 exposed at (1) preferred temperature (Tpref) at 24 °C; (2) low temperature (LT) at 21 °C; (3) either individuals without treatment (Control) with fluctuating temperature of the habituation site, to compare the effect on the viability, concentration, and integrity of DNA, as well as the presence of the cytoplasmic droplet (CD). Sperm viability decreased by approximately 50% in organisms exposed to Tpref, accompanied by a significant reduction in concentration. Likewise, the CD evaluation showed a retention percentage of 30% at the cauda level, while the LT and Control groups showed 5% in this terminal area of the epididymis. The percentage of sperm with intact DNA was also decreased in the Tpref group compared to the LT and control groups. These findings highlight how thermal environments impact reptilian reproduction, reinforcing not only what has already been found in these species in recent years, but also the repercussions that it presents in the medium- or long-term exposure in these organisms at an ecological level, as well as the consequences with the potential damage and even infertility, in case of prolonged exposition to preferred temperatures.

## Simple Summary

Regional climate changes caused by global warming could particularly affect the development, behavior, and reproduction of behavioral thermoregulators. Although lizards require a defined temperature range (preferred temperature) to carry out their physiological and metabolic activities, the temperatures for gonadal development and sperm production should be lower than those for behaviors such as foraging or predator avoidance; this could lead to problems in sperm maturation, as has been reported in other species. We describe the effect of temperatures on sperm parameters in the lizard *Lepidophyma gaigeae* and the optimal range that favors them, which is lower than the behavioral optimum. As global warming increasingly impacts species and ecosystems. Our findings inform environmental and species management with respect to male reproductive capacity, which could increase the risk of extinction in some lizard species.

## 1. Introduction

Under the thermal dependence of physiological processes, ectotherms must regulate their body temperature close to their preferred temperature range (Tpref) for the optimal performance of various physiological functions, such as metabolic rate, locomotor performance, digestive efficiency, growth rate, egg production, spermatogenesis, and sperm maturation [1,2,3,4,5]. To this thermal dependence, reports on the susceptibility of germ cell damage to extreme temperature changes in lizards date back as far as the 1940, showing that high temperatures cause sterility [6] and subsequently, asserting that there is a greater sensitivity of germ cells concerning somatic cells under conditions of temperature stress [7].

A study on *Sceloporus siniferus* [8] revealed the possibility of a negative effect of temperature on epididymal sperm maturation due to CD retention as the collection of males between 2015 and 2016 shows a significantly different percentage of sperm with CD, being 10% in those collected in 2015, which showed a cloacal temperature of 28 °C and 40% in those collected in 2016 with a cloacal temperature of 34 °C.

Studies on *Sceloporus aeneus* detail the positive and negative repercussions of subjecting specimens to preferred temperatures, highlighting locomotor performance, and sperm quality. In the first case, animals that are kept in a preferred temperature environment favor motility interval, sprint speed, and foraging; however, some sperm parameters are decreased, such as concentration, viability, and motility, as well as the increase in spermatozoa with CD [3]. These aspects have been considered by Méndez-De La Cruz et al. (2014) [2], who proposed that low temperatures, also given by the inactivity schedules of the organisms, may be in charge of favoring such reproductive parameters.

Recently, in *Sceloporus megalepidurus*, these aspects were shown, where temperatures below (28–30 °C) the range of preferred temperature (33.64 ± 3.20 °C) favor viability and epididymal sperm maturation, contrary to individuals subjected to higher temperatures (33.6–36 °C), where the cases of sperm DNA fragmentation and the presence of CD increased [5].

Even more, in *Lepidophyma pajapanense*, it was found that copulation occurs in the coldest months and ovulation at the beginning of the warmest season; perhaps that asynchrony in reproduction is due to the favorable temperature for males’ mismatch with the ones that are favorable for the fecundation. Fertilization is achieved because these lizards with an asynchronous reproductive cycle have sperm storage. Moreover, a significant percentage of infertile eggs were found, perhaps due to low sperm quality or to the long sperm retention in females, or both [9]. Therefore, the interaction between sperm quality and temperature is an important issue to resolve.

The study of the thermal ecology of lizards is important for understanding their distribution, life cycle, ecology, and reproductive aspects. It also assesses the risk of extinction in the face of climate change and its general consequences. Furthermore, it reveals how species can reach physiologically active body temperatures even in thermally unfavorable environments.

Also known as Gaiger’s lizard [10], *Lepidophyma gaigeae* is an endemic species of Mexico, whose habitat includes pine, and oak forests, grasslands and xerophytic scrub preferably in outcrops and between the crevices of limestone rocks [11], at an altitude of 1500 to 2500 m, in states such as Hidalgo and Querétaro [12]. It is a small-sized viviparous lizard, between 47 and 54 mm (snout-vent length)—one of the smallest species of the genus [13]. It presents a diurnal activity period [14], although some nocturnal individuals have been found [13]. In areas of thermal ecology, Arenas-Moreno et al. (2018) [13] state that *L. gaigeae* has a preferred temperature range of around 24 °C (23 °C minimum, 27.4 °C maximum), a range similar to that reported in other species of its genus [15,16].

The present study aims to determine if the preferred temperature could be the cause of the alteration in some sperm parameters in the testis and/or epididymis, as well as the migration of CD and the effect of DNA integrity in spermatozoa of the lizard *L. gaigeae*.

## 2. Materials and Methods

As a species under special protection according to NOM-059 Mexican Official Standard, SEMARNAT, México City, México, 2010 [17]. and so as not to alter the availability of adult males in the area and consider their distribution in the future, only 12 male individuals were captured during December 2020 in the surroundings of Landa de Matamoros, Querétaro, México, in the area known as “El Lobo” (21°17′32.5″ N 99°07′13.1″ W), with a collection schedule from 9:00 h to approximately 15:00 h, which coincides with their diurnal usual activity schedule. The collection permit SGPA/DGVS/02523/19 was granted to Dr. Fausto Roberto Méndez de la Cruz by the Undersecretariat of Management for Environmental Protection and the General Directorate of Wildlife, a dependency of the Ministry of Environment and Natural Resources (SEMARNAT).

For the selection, male lizards with snout-vent length (SVL) greater than 47 mm and a visible bulge in the ventral region due to the presence of hemipenes inside the cloaca, characteristics described by Goldberg and Camarillo (2003) [18].

The specimens were captured by direct capture methods, either manually or with the use of a fishing rod, depending on the location of the specimens and the availability of space for the free collection of the specimens. In the case of capture with a fishing rod, the subject is caught by the neck by the loop of silk, where its attempt to escape holds the instrument’s line together with its scales, facilitating the grip without suffocating the animal.

The captured animals were housed in 20 × 15 cm blanket bags, with a maximum of four specimens each, to be later contained in plastic racks to facilitate their transfer and to be transported to the vicinity of the Laboratory of Morphophysiology and Biochemistry of Spermatozoa, Universidad Autónoma Metropolitana-Iztapalapa, to proceed with the acclimatization procedures. The transport vehicle maintained a temperature between 21 °C and 24 °C according to the preferred temperature range of the species (23 °C minimum–27.4 °C maximum) [13], avoiding stretches that generate sudden movements during their arrival at the facilities to reduce levels of stress in the animals like auditory stress, decreased or increased body temperature, hyperactive shock syndrome, capture syndrome, among others. The habituation of the specimens before being sacrificed (in the case of the control group) or subjected to acclimatization (Tsel and LT groups) had a period of 36 h.

### 2.1. Inclusion of Specimens

The lizards were separated into three groups (four individuals per group): control group, analyzed without treatment with fluctuating temperature of the habituation site without reaching the upper limit of its minimum critical temperature (17.6 °C) [13]; preferred temperature (Tpref) (24 °C), and low temperature (LT) (21 °C), following the considered observations of [2,3,5,18]), where a range between the lower limits of the Tpref and without reaching the upper limit of the critical minimum temperature of the species is considered. Treatments were maintained during the activity time of the lizards (9:00 h to approximately 15:00 h), placed in incubators “Hova Bator 1602N” of thermal radiation, with the required temperature for Tpref and LT, and without thermal radiation for the control group, including the temperature during scotophase [13]. They were checked daily and maintained with water and live food (crickets and mealworms) ad libitum during the habituation period in the control group and after 14 days in the acclimatization groups.

### 2.2. Collection of Biological Material

The control specimens were sacrificed by intraperitoneal injection with 0.10 mL of sodium pentobarbital (Sedalpharma, from Pet’s Pharma de México, S.A. de C. V) 36 h after the arrival of the specimens at the University facilities for the control group and after 14 days of treatment for the remaining groups, with adherence to the guide for the care and use of laboratory animals (Institute of Laboratory Animal Resources, National Research Council, 1996), as well as the NOM-033-SAG/ZOO Mexican Official Standard Methods for killing domestic and wild animals, PROFEPA, México City, México, 2014 [19]. Both testes and epididymides were extracted, measured (diameters and lengths) with a digital caliper (precision = ±0.5 mm), and weighed with a METTLER TOLEDO AB204-S balance (METTLER TOLEDO, Columbus, OH, USA) (precision = ±0.1 mg), of which, those on the left side were used for sperm parameters, although those on the right side were fixed in 4% paraformaldehyde (PAF) (Thermo Scientific, Waltham, MA, USA) and subsequently sagittally sectioned for further study. Each epididymis was sectioned into three regions with similar proportions: caput, corpus, and cauda [5].

To obtain sperm parameters, the organs were placed in a four-well Petri dish with 250 μL of physiological Ringer’s solution (NaCl, KCl, KH_2_PO_4_, and CaCl_2_ 2H_2_O from Baker Laboratories, Phillipsburg, NJ, USA) to obtain as many spermatozoa as possible. Then the biological material was transferred to a 1.5 mL Eppendorf tube [20]. Subsequently, the material was centrifuged at 250 G for 5 min, the supernatant was removed, and the cell button was disaggregated with 250 μL of Ringer’s solution twice. Thus, the resultant will be “washed spermatozoa”.

The spermatozoa obtained were evaluated according to the World Health Organization specifications for the evaluation of human semen (2010, WHO) with necessary adjustments for lizard cells obtained from the different organs. The variables and methods for obtaining them were as follows:

Total number of spermatozoa per organ (n spz × 10^6^/organ): obtained by counting the spermatozoa with the Neubauer chamber, making a dilution depending on the density of spermatozoa identified at a field of 40×, filling both chambers with 10 to 15 µL of the dilution.

To obtain the sperm concentration, the following formula was used: N/n × (1/20) × dilution factor, where N is the total number of spermatozoa counted in both chambers and n is the number of squares counted.

To calculate the viability percentage and CD, a 5 µL aliquot of the sperm suspension was placed on a slide and mixed with an aliquot of the same volume of Spermavit supravital stain, then smeared and observed under brightfield microscopy for counting.

Percentage of DNA integrity: A 10 µL aliquot of the sperm suspension was placed on a slide to make a smear and allowed to dry at room temperature, then included in Carnoy’s solution (ethanol from Meyer Laboratories, Tláhuac, México City, México, chloroform, and acetic acid from Baker Laboratories, Phillipsburg, NJ, USA) for approximately 24 h in a cool, dark place. Afterwards, the fixative was removed and the slides were included in acridine orange solution for 5 min. Then, the excess dye was removed and left to dry isolated from light. Finally, the slides were analyzed with the aid of fluorescence microscopy at 40×.

The handling of chemical–biological waste generated in the experiment was instructed by the NOM-033 Mexican Official Standard, Safety conditions for working in confined spaces, STPS, México City, México, 2015 [21].

### 2.3. Statistical Analysis

Statistical analysis was used for the evaluation of the three treatments, as well as to evaluate variability and random errors so that possible environmental and temporal effects are distributed equally among the treatments, followed by a Tukey post hoc test. The data were previously analyzed to meet the assumptions of the parametric test used (normality and homoscedasticity), considering the data from the different organisms analyzed as independent.

## 3. Results

According to the criteria [13], adult male individuals were collected. Presenting an SVL of 51.60 ± 2.17 and a weight of 4.37 ± 0.33, similar to the reported adult size.

**Figure 1:**
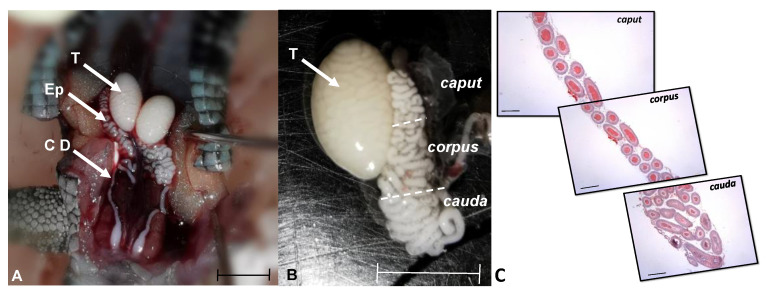
Position and regionalization of male gonads of *L. gaigeae* in the reproductive stage. In (**A**): Location of the gonadal position. Arrows show the testis (T) and epididymis (Ep) of the specimen. In (**B**): Testis and epididymis with regionalization to identify the area of the caput, corpus, and cauda. Bar: 0.5 cm. In (**C**): Photomicrographs in sequence showing the different histomorphology of the three epididymal regions in longitudinal section, obtained with H&E at 5×. Bar at 50 µm.

After the period determined for the corresponding study, the organs of interest were obtained (Figure 1). It should be noted that all specimens presented spermatozoa in the testis and epididymis; however, those organs of the individuals that were subjected to some temperature treatment (24 °C and 21 °C) presented lower testicular weight and length.

### 3.1. Gonadal Size and Weight

**Figure 2:**
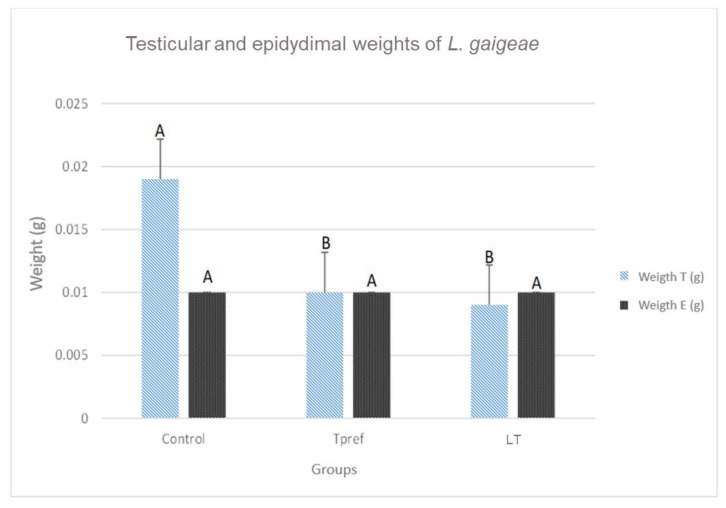
Weight (g) of testis and epididymis of *L. gaigeae* specimens with and without treatment at different temperatures. The bars indicate the distribution of the mean value of the data obtained from each treatment and region. Different letters indicate significant differences between the comparison of groups, according to the analysis of variance (ANOVA) and the Tukey post hoc test, given with a *p*-value < 0.05.

**Figure 3:**
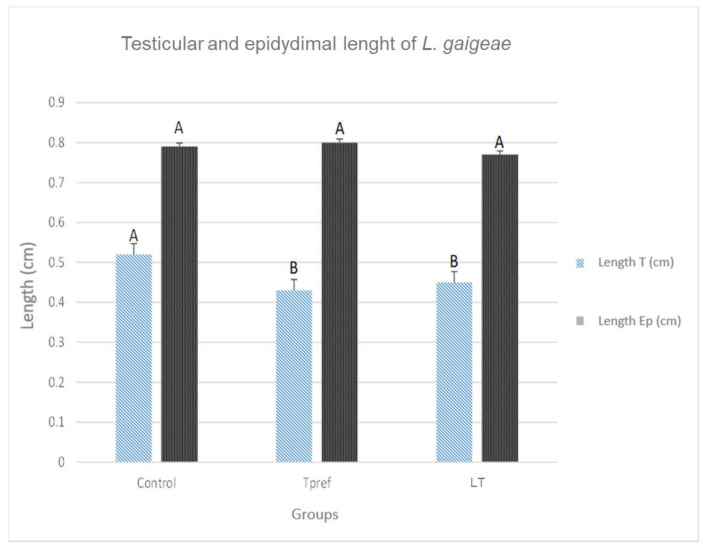
Testicular and epididymal lengths of *L. gaigeae* specimens with and without treatment at different temperatures. The bars indicate the distribution of the mean value of the data obtained from each treatment and region. Different letters indicate significant differences between the comparison of groups, according to the analysis of variance (ANOVA) and the Tukey and Kruskal–Wallis post hoc test, given with a *p*-value < 0.05.

The records obtained show a lower testicular weight in the groups that were subjected to some temperature treatment (24 °C and 21 °C) (Figure 2), either preferred or low (0.010 ± 0.003 and 0.009 ± 0.001, respectively), as well as their length (0.43 ± 0.07 and 0.45 ± 0.04, respectively) (Figure 3), compared to the control group. Despite this, the data obtained did not show significant differences in the epididymis.

**Figure 4:**
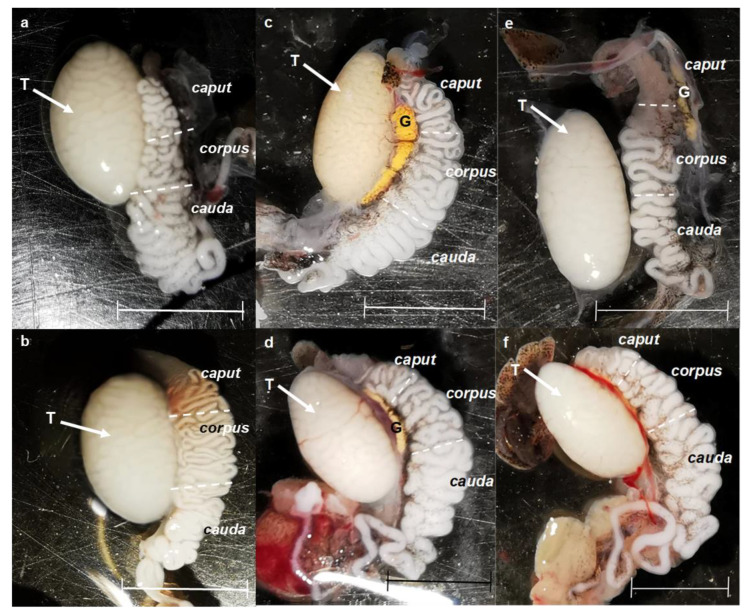
Physical variations in the testis and epididymis were found in some individuals of *L. gaigeae* subjected to different temperatures. Images (**a**,**b**) correspond to organs collected in the control group; images (**c**,**d**) correspond to organs collected in the preferred temperature treatment (Tpref); images (**e**,**f**) correspond to organs collected in the low temperature treatment (LT). G: Fat body. T: Testis. Bars at 0.5 mm.

Although testicular morphology was similar between the groups, it was found that one of the individuals treated at low temperature showed an apparent decrease in the concentration of spermatozoa in the caput area (Figure 4, image e) in conjunction with two subjects who appeared to have a greater amount of blood vessels between the two organs of interest (Figure 4, image f).

### 3.2. Sperm Parameters

#### 3.2.1. Sperm Concentration

There are significant differences between groups: gonadal size, weight, and sperm concentration ratified the decrease between the regions analyzed and between groups, both in the testis and epididymis in the specimens with temperature treatment (24 °C and 21 °C).

The lower sperm concentration presented in the Tpref (24 °C) and LT (21 °C) treatments is manifested from the testicular region (25.3 ± 12.74 spz × 10^6^ and 30.6 ± 6.60 spz × 10^6^, respectively) and continues in the same way in the epididymis (Table 1). It should be noted that, as described in Figure 4b, a specimen treated at LT (21 °C) presented a lower sperm concentration at the caput level (10.6 ± 0.81), which could be corroborated in the quantitative analysis and could provide a significant difference in the comparison between groups.

On the other hand, the groups treated at Tpref (24 °C) and LT (21 °C) showed similarities in corpus (19 ± 4 and 15.6 ± 4.27, respectively) and cauda (21.6 ± 3.05 and 23.6 ± 6.13, respectively) epididymis, however, having significant differences in comparison with the control group (38 ± 7.93 in corpus and 39 ± 9.53 in cauda).

It should be noted that the present study of sperm concentration is the first to show a quantitative analysis of the species in its reproductive stage, which could be very close to what would be found in natural conditions, as well as the impact that temperature would have on its activity period.

#### 3.2.2. Sperm Viability

**Figure 5:**
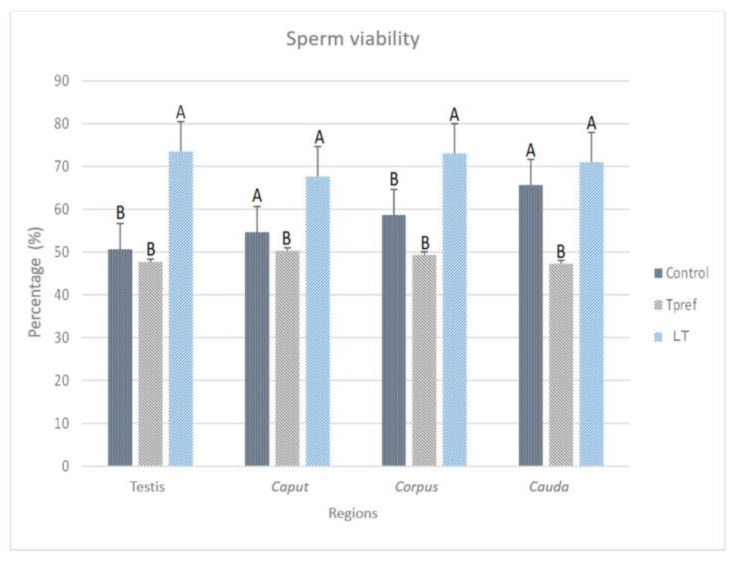
Percentage of sperm viability of *L. gaigeae* with and without treatment at different temperatures. The bars indicate the distribution of the mean value of the data obtained from each treatment and region. It is evident that the maintenance of the viability percentage in the groups with low temperature treatment (21 °C) and the increase in sperm viability in the control group, compared to the remaining group, whose values decrease notably and maintain a viability of 50%. Different letters indicate significant differences between the group comparisons, according to the analysis of variance (ANOVA) and Tukey’s post hoc test, given with a *p*-value < 0.05.

**Figure 6:**
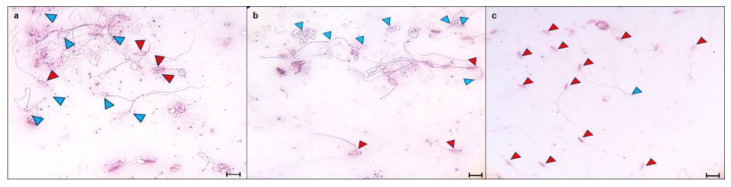
Representative photomicrographs of sperm viability of *L. gaigeae* with and without treatment at different temperatures stained with eosin–nigrosin for viability assessment with the plasma membrane dye penetration method, obtained by brightfield microscopy. (**a**): Control group; (**b**): LT group; (**c**): Tpref group. Blue arrowheads: viable sperm; red arrowheads, non-viable sperm. ×100 Bars corresponding to 10 µm.

The sperm viability analysis (Figure 5), obtained by the eosin–nigrosin staining method (Figure 6), shows the impact of temperature treatments on these parameters. Contrary to the analysis of sperm concentration, significant differences between groups were found in one of them, especially at the epididymis level. The results stand out in different points, highlighting at least three important aspects to be considered.

Firstly, it should be noted that the percentage of sperm viability in the control group and LT (21 °C) is above 60% at the cauda level. In this case, the percentage of viable spermatozoa in the control group ranges from 50.66% at the testicular level to 65.6% in the cauda.

Secondly, it is important to mention that the group treated at LT (21 °C) showed a viability percentage ranging from 73% to 71%, even though, at the caput level, the count is 67.6%. No differences were found between the control and Tpref (24 °C) groups. It is worth mentioning that it was in the Tpref group (24 °C) where the individuals showed a lower sperm concentration, mainly in the caput of one of them. A maximum of 50.3% in caput, but lower in the remaining regions—up to 47.3% in cauda. Even though the impact may not be drastic compared to that found in other species [3,5]. The comparison with the concentration and other parameters corroborates the effect at the reproductive cellular level.

#### 3.2.3. Cytoplasmic Droplet Percentage

**Figure 7:**
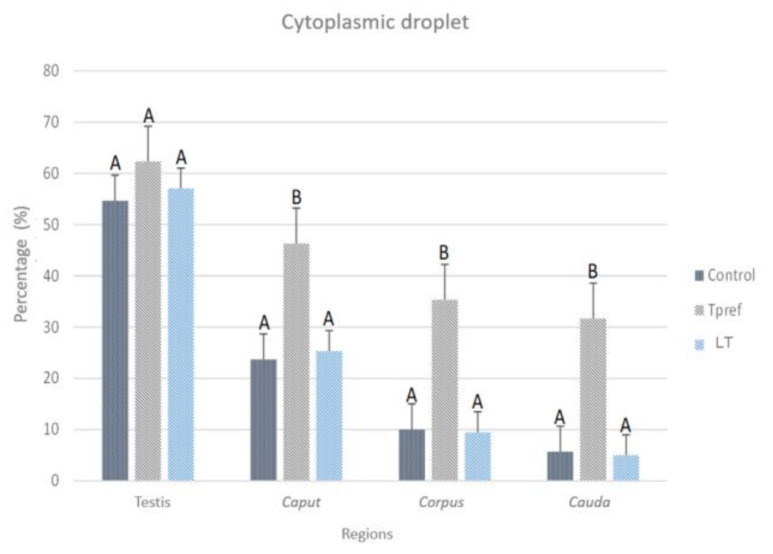
Percentage of spermatozoa with cytoplasmic droplet (CD) of *L. gaigeae* with and without treatment at different temperatures. The bars indicate the distribution of the mean value of the data obtained from each treatment and region. Evidence of CD removal at the testicular level is shown in the control group and with low temperature treatment (21 °C). All groups showed a decrease in CD as they moved to the caput zone, with a difference in the percentage of spermatozoa found with this characteristic. Different letters indicate significant differences between the comparison of groups, according to the analysis of variance (ANOVA) and Tukey’s post hoc test, given with a *p*-value < 0.05.

**Figure 8:**
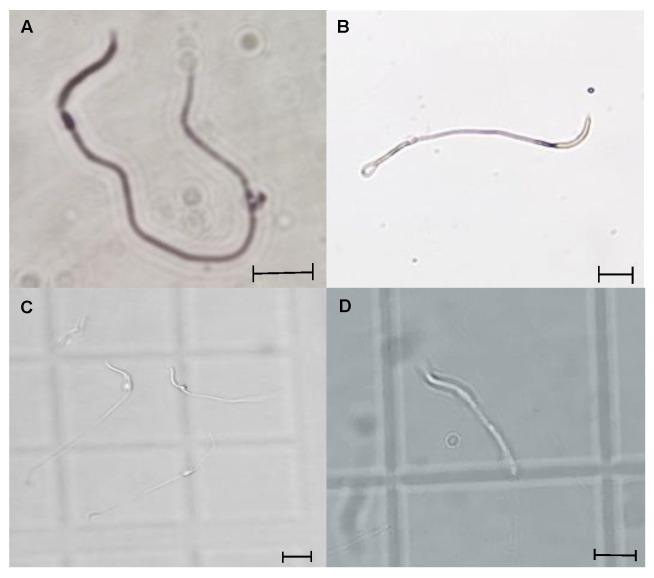
Representative photomicrographs of *L. gaigeae* spermatozoa stained with eosin–nigrosin (**A**,**B**) and unstained (**C**,**D**) for evaluation of the presence of cytoplasmic droplet (CD) by brightfield microscopy. (**A**,**C**): Spermatozoa with CD. (**B**,**D**): Spermatozoa without CD. ×400. Bars indicate the dimensions of the photomicrographs, corresponding to 10 µm.

The evaluation of CD in the spermatozoa obtained from the testis and epididymis (Figure 7) was carried out considering only the cells that presented the shape of a fully formed spermatozoa and without excess of cytoplasmic residues in the head (Figure 8). Only the spermatozoa counted were found with the CD in the neck of the cell. Thus, the Tpref group showed a higher retention of CD in the last epididymal region compared with the control and the LT (21 °C) group. The values ranged from 54% and 57% in the testis, respectively, to around 5% of sperm with CD in the control and LT groups at the cauda level. Although the Tpref (24 °C) group also presented a lower percentage of CD retention, this remained above 30% in the last epididymal region, despite not showing significant differences at the testicular level compared with the two groups mentioned.

#### 3.2.4. DNA Integrity

**Figure 9:**
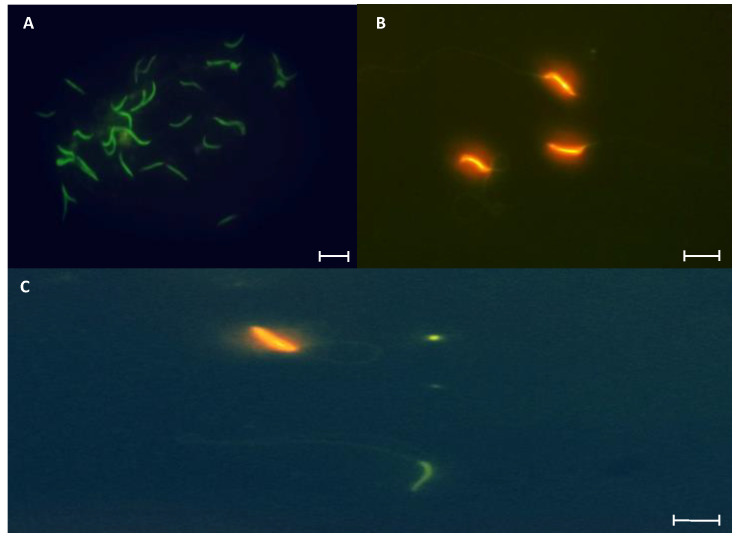
Representative photomicrographs of *L. gaigeae* spermatozoa stained with acridine orange for DNA integrity evaluation by fluorescence microscopy. (**A**): Spermatozoa with intact DNA. (**B**): Spermatozoa with fragmented DNA. (**C**): Indicates the bipolarity of the staining found in the cell samples. ×400. Bars at 10 µm.

**Figure 10:**
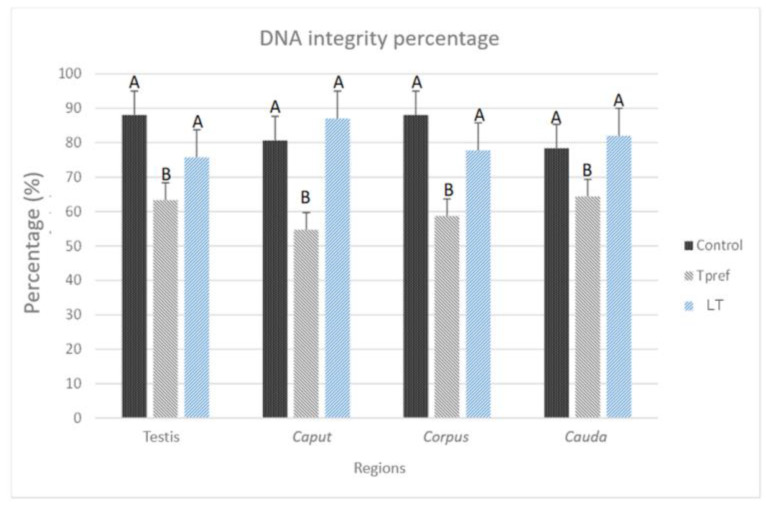
Percentage of DNA integrity of *L. gaigeae* spermatozoa with and without treatment at different temperatures. The bars indicate the distribution of the mean value of the data obtained from each treatment and region. The percentage of spermatozoa with intact DNA is maintained, except for the group with treatment at the preferred temperature (Tpref) in all the areas evaluated, which seems to be increasing, shortening the difference between the remaining groups. Different letters indicate significant differences between the comparison of groups, according to the analysis of variance (ANOVA) and Tukey’s post hoc test, given with a *p*-value < 0.05.

The projected staining after treatment with acridine orange, where spermatozoa with intact DNA are cataloged if they manifest a green hue at the level of the cell head, while those presenting an orange hue were counted as cells with fragmented DNA (Figure 9) allowed for the demonstration of the differences in terms of variation between groups and regions (Figure 10). In the first case, the control group remained at a relatively constant interval, with nearly 80% found in practically all regions, with the caudal region of the epididymis being the area where the lowest percentage of spermatozoa with intact DNA was found. Despite this, the average recorded was 78.3%.

On the contrary, the group treated at Tpref (24 °C) exhibits a lower percentage of spermatozoa with intact DNA from the testicular level (63.3%), whereas in caput presents its lowest percentage value (54.6%) which subsequently increases in the corpus and cauda (58.6% and 64.3%, respectively). This finding contrasts directly with the LT group (21 °C), whose case is similar to that found in the control group, with its percentage in the caudal region even higher than in the last mentioned (82%).

## 4. Discussion

The formation of spermatozoa takes place in the seminiferous epithelium of the testis through a process known as spermatogenesis, a process by which, from diploid round cells (spermatogonia) located adjacent to the basement membrane of the seminiferous tubules, they differentiate until forming spermatozoa, advancing towards the tubule lumen [22,23]. However, even when the morphological differentiation of the spermatozoa culminates in the testis when the spermatozoa are released from it, they have not yet acquired the capacity to fertilize the oocyte; for this reason they need to pass through the epididymis where they acquire this capacity, a process known as epididymal sperm maturation [24,25]; the following stand out: the loss of cytoplasmic droplet (CD) and chromatin hypercompaction about the maintenance of DNA integrity [8,26,27].

Climate change may cause the extinction of species, as well as a shift in their distribution in the coming decades [2,28]. Since 1975, 12% of local lizard populations have become extinct, as well as 4% around the world. By 2080, extinction is projected to approach 39% globally, and local species extinction by 20%. If the maximum temperature derived from global warming continues unchanged, it is estimated that, in Mexico, in the year 2050, 56% of the viviparous species will become extinct and 66% by 2080. The causes of extinction may be attributed to low thermal quality (thermal conditions that favor the lizards’ survival, which, if adverse, force the individual to restrict its activity schedule), impairing the lizards’ ability to perform basic biological activities and increasing their restricted hours [28]. Nevertheless, the temperatures may decrease the thermal quality of the sperm (the temperature at which sperm cells function properly, meeting the conditions for fertilization), and the males become infertile. The results of this study show that the sperm is labile (in quality and morphology) when lizards are overexposed to the preferred temperatures and is even worse at higher temperatures.

For example, as occurred with the difference in testicular size, previous studies with *Sceloporus megalepidurus* [5] showed that individuals who remained in treatment at the preferred temperature for 14 days presented smaller testes and epididymal, which was one of the first characteristics described in reptiles affected by temperature changes. It is known that the volume of reproductive organs can be an indirect indicator of sperm concentration [29]. In the present study, we found that this difference in volume could be attributed to the decrease in spermatozoa in the group exposed to preferred temperature [3,22,24]. In the *Lepidophyma pajanapense*, a high percentage of infertile eggs was found [29]. That low percentage could be attributed to the long sperm retention or the low quality of the sperm cells, even when copulation occurs during the coldest months of the year, perhaps due to the inability to manage the warm temperatures during the ovulation months.

It is known that chronic hyperthermia stress can cause alterations in spermatogenesis [30], as has also been shown in mammalian species such as rats [31] and sheep [32]. In addition, these thermal conditions tend to influence reproductive conditions from the hypothalamus–pituitary–gonad axis through the increase in glucocorticoids and the repression of gonadotropin-releasing hormone (GnRH), fundamental for the promotion of spermatogenesis and testosterone production [33], also causing a reduction in the number of Leydig cells as well as spermatogonia in the seminiferous tubule [34].

The integrity of the plasmatic membrane is one of the most important characteristics to consider for the evaluation of viable spermatozoa [35]; therefore the use of the eosin–nigrosin dye allowed for the demonstration of the decrease in this characteristic in individuals maintained at preferred temperatures, bringing it below 50%, a value resembling those shown in other reptile species [3,5]. On the other hand, knowing that sperm viability in each of the epididymal regions may be different according to the functionality of its cellular structure and the achievement of non-functional sperm absorption [36], the coincidence in the percentage, even in the four regions evaluated, allows us to question whether the problem may even come from the concentration of androgens. Given that the epididymis is a hormone-dependent organ (mainly dihydrotestosterone [37]), different analyses have coincided in that the lack of these contributes in the decrease in epididymal weight, as well as in the regulation of secretions that reach the luminal region, such as proteins and transcription, are factors important for sperm viability and flagellar motility mainly [13,38,39,40,41,42,43,44]. In addition, reduction in epididymal epithelium volume may also occur as a consequence of the effect of temperature, with increased apoptosis of constituent cells, such as principal cells [45].

Although, once again, the effect that CD causes in lizard spermatozoa has not been analyzed in depth, it is clear with the present study, as well as those reported [3,5,8], that thermal modifications tend to influence its presence along the different regions of the epididymis. This coincides with numerous reports in mammals that document the increase in CD under conditions of testicular hyperthermia, indicative of immature sperm, given by changes in ambient osmolarity or decreased antioxidant system and increased reactive oxygen species (ROS) and plasma membrane lipid peroxidation [25,46,47,48].

However, some studies have raised the possibility of a protective function in the face of unfavorable conditions for spermatozoa [49], showing that CD spermatozoa may have a higher tolerance to heat stress. In addition to this, it is known that CD has the presence of aquaporins, which can collaborate with osmotic adaptation, migration, or sperm storage [50], in addition being enzymatically active [51] and an important source of energy for the different maturational aspects of the cells, which is why they can remain in the cell throughout the epididymis [52].

The relationship that CD has had around the exposure of organisms to high temperatures is given in many cases by its relationship with lipid droplets (LDs), which, although in some cases, supplements the function of the latter to provide energy [53], can also cause lipoperoxidation of the plasma membrane by increasing ROS given mainly by the decompensation of antioxidant enzymes. In hyperthermia treatments, GL has been found along Leydig cells, important for testosterone production and sperm maintenance [48], but mostly involved in the breakdown of disulfide bridges involved in DNA compaction [54]. The relationship of increased ROS with sperm viability and morphology also derives from the cell death by apoptosis, where studies show the activation of caspase 3 in seminal samples with high DNA fragmentation index [54].

Lizards around the world are facing problems in surviving; one of the most important is global warming. This study determines that warming temperatures affect not only the hours of activity but also the fertility of males. It was found that rising temperatures arrested female fertility during the El Niño Southern Oscillation event in *Sceloporus mucronatus* [55] (Rodríguez Romero and Méndez de la Cruz, 2004). This study determines that sperm quality could be severely affected by preferred temperatures and may be the cause of infertility. Therefore, the decline of populations may also be due to the infertility of males exposed to warmer temperatures. Furthermore, it may promote dissociation in reproductive cycles, as in *L. pajapanense*, where males copulate during the colder months while females ovulate in the warmer ones [9]. This study shows that the conditions for fully fertile males are an issue that demands more studies as there is a connection between physiology and environmental temperatures.

## 5. Conclusions

The present study has shown the effect of temperature on *Lepidophyma gaigeae* spermatozoa, given by the controlled thermal maintenance that emulates different environmental conditions in the organism, showing that the temperature at which the individual tends to perform better in terms of ethological and physiological parameters tends to be detrimental in reproductive terms, causing a decrease in gonadal size, concentration, viability, and probably spermatic maturation. Furthermore, we demonstrate that temperatures below the preferred temperature maintain not only sperm quality but also DNA integrity.

## Figures and Tables

**Table 1:** Sperm concentration of *L. gaigae* with and without treatment at different temperatures. Data represent n10^6^ spz/organ. Asterisks indicate significant differences between group comparisons, according to analysis of variance (ANOVA) and Tukey’s post hoc test, given at *p*-value < 0.05.

	Testis	*Caput*	*Corpus*	*Cauda*
Control	80 ± 8.88	18 ± 2	38 ± 7.93	39 ± 9.53
Tpref	* 25.3 ± 12.74	14.3 ± 3.51	* 19 ± 4	* 21.6 ± 3.05
LT	* 30.6 ± 6.60	* 10.6 ± 0.81	* 15.6 ± 4.27	* 23.6 ± 6.13

## Data Availability

We hereby declare the open accessibility of data from our research for transparency purposes. Due to time and technical status, our data is not yet available on Figshare to share DOI; however, we are committed to uploading it as soon as possible. For now, we can share them, if you wish, via email.

## Supplementary Materials

The following supporting information can be downloaded at: https://www.mdpi.com/article/10.3390/ani15121784/s1.

## Abbreviations

CD	Cytoplasmic droplet
LT	Low temperature
Tpref	Preferred temperature
ROS	Reactive oxygen species
SVL	Snout-vent length
LD	Lipid droplet
