# Supplementary material for: Temperatures Lower than Preferred Ones Maintain DNA Integrity and Sperm Quality of Lepidophyma gaigeae (SQUAMATA: XANTUSIIDAE)
Source: Animals (Basel). 2025 Jun 17;15(12):1784. doi: 10.3390/ani15121784 (PMC12189620; doi:10.3390/ani15121784)
Supplement: Supplementary file 1 [file animals-15-01784-s001.zip › animals-3610617-supplementary.pdf]

Casa abierta al tiempo

UNIVERSIDAD AUTÓNOMA METROPOLITANA-*Iztapalapa*
COMISIÓN ACADÉMICA DE ETICA DE LA DIVISIÓN
DE CIENCIAS BIOLÓGICAS Y DE LA SALUD

Ciudad de México a 29 de octubre de 2021
Dictamen: CECBS21-04

A quien corresponda:

La Comisión Académica de Ética de la División de Ciencias Biológicas y de la Salud, conforme a sus competencias revisó el protocolo de Investigación de la alumna **Daniel Uriostegui Escoto** titulado: **"EFECTO DE LA TEMPERATURA EN LA ORGANIZACIÓN HISTOLÓGICA DE TESTÍCULO Y EPIDÍDIMO Y EN LA PRESENCIA DE INDICADORES DE MADURACIÓN ESPERMÁTICA DE LA LAGARTIJA *Lepidophyma gaigeae*",** dirigido por la doctora: **Edith Arenas Ríos.**

Encontrando que dicho proyecto**, SI CUMPLEN CON LOS LINEAMIENTOS PARA LA CONDUCCIÓN ÉTCA DE LA INVESTIGACIÓN, LA DOCENCIA Y LA DIFUSIÓN EN LA DIVISIÓN DE CIENCIAS BIOLÓGICAS Y DE LA SALUD DE ESTA INSTITUCIÓN.**

Cabe recordar que dichos lineamientos derivan de la legislación mexicana que atañe a la parte bioética de nuestro quehacer.

Se extiende la presente para los fines que convengan al interesado

ATENTAMENTE
Casa Abierta al Tiempo

DRA. MARÍA DEL ROSARIO TARRAGÓ CASTELLANOS
Presidenta

c.c.p. archivo
